# Molecular Docking of Cryptoconcatones to α-Tubulin and Related Pironetin Analogues

**DOI:** 10.3390/plants12020296

**Published:** 2023-01-08

**Authors:** Gérard Vergoten, Christian Bailly

**Affiliations:** 1 Institut de Chimie Pharmaceutique Albert Lespagnol (ICPAL), Faculté de Pharmacie, University of Lille, 3 rue du Professeur Laguesse, BP-83, F-59006 Lille, France; 2 OncoWitan, Consulting Scientific Office, Wasquehal, F-59290 Lille, France

**Keywords:** anticancer agents, *Cryptocarya* species, cryptoconcatones, pironetin, tubulin binding

## Abstract

Cryptoconcatones A-L represent a series of 12 dihydropyrone derivatives isolated from the evergreen tree *Cryptocarya concinna* Hance, which is well distributed in southeast Asia. The lead compound in the series, cryptoconcatone L, has revealed antiproliferative activity against cultured cancer cells but its mechanism of action remains unknown. Based on a structural analogy with the anticancer natural product pironetin, which is well known for binding covalently to α-tubulin and for functioning as a microtubule polymerization inhibitor, we investigated the interaction of cryptoconcatones with tubulin dimers using molecular docking. The α-tubulin binding capacity of each compound was quantified (through calculation of the empirical energy of interaction Δ*E*) and structure–binding relationships were delineated. Two compounds were found to interact with α-tubulin much more potently than pironetin: cryptoconcatones F and L. In both cases, the facile formation of a covalent bond with Cys316 was evidenced, as observed with the parent compound pironetin. A few other pironetin analogues were investigated, including spicigerolide, which is an analogue of another known α-tubulin binder. Altogether, this study points to the identification of a series of 5,6-dihydro-α-pyrones as α-tubulin-binding agents. The study contributes to a better understanding of the mechanism of action of cryptoconcatones and should help the design of analogues targeting the pironetin site of α-tubulin.

## 1. Introduction

Microtubules are elongated cytoskeletal structures formed from the polymerization of tubulin dimers. They delimit a dynamic tubular network subjected to continuous cycles of polymerization and depolymerization. These biopolymers are made of α-tubulin and β-tubulin heterodimers. For many years, microtubule-targeting agents (MTAs) have represented effective drugs for cancer treatment. Different classes of MTAs are used to treat solid tumors, principally taxanes (paclitaxel, docetaxel, cabazitaxel), vinca-alkaloids (vinorelbine, vincristine), and a few others (epothilone, eribulin) [1,2]. They represent potent cytotoxic agents which are useful for blocking tumor cell growth, and display additional key properties such as decreasing angiogenesis and cell migration, reducing metastasis, and activating innate immunity to promote a proinflammatory response [3]. Most of these drugs were discovered more than 50 years ago, but they remain widely used daily in cancer therapeutic units. Some of these drugs, notably taxonoids, have “changed society”, as underlined recently [4]. However, the use of these compounds is limited by significant toxicities (notably peripheral neuropathies) and the development of multidrug resistance. These limitations can be reduced upon combination with targeted molecules, such as kinase inhibitors or immune checkpoint inhibitors, but nevertheless the search for novel MTAs is needed to improve cancer treatments [2]. Novel anti-tubulin drugs continue to be designed and evaluated experimentally [5,6].

**Figure 1:**
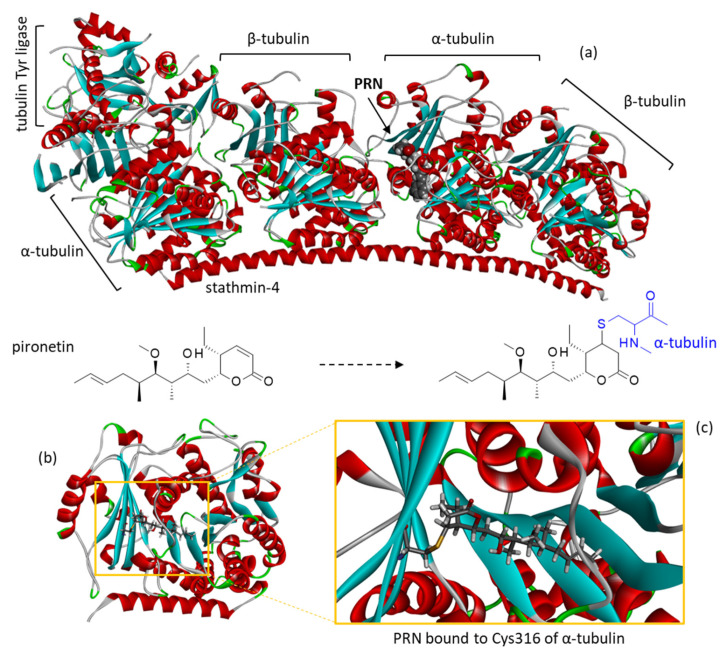
The molecular model of pironetin (PRN) bound to β-tubulin. (**a**) A docking model of PRN binding to the α/β-tubulin dimer interacting with stathmin-4 and tubulin tyrosine ligase (PDB access code: 5FNV) was built. The pironetin active site of α-tubulin is highlighted. (**b**) A close-up view of PRN bound to the active site. (**c**) Detailed view showing the covalent bond between the lactone unit of PRN and the thiol group of Cys316 residue, as indicated on the corresponding structures.

All clinically approved MTAs interact with the β-tubulin component of tubulin heterodimers. Several drug-binding sites have been identified on β-tubulin, such as the vinca site, maytansine site, taxane site, colchicine site, and laulimalide/peruloside site [7]. Recently, another site at the junction of α- and β-tubulin was identified for cyclodepsipeptide gatorbulin-1, isolated from marine cyanobacteria [8]. There is only one known binding site within the α-tubulin component, the so-called pironetin site (Figure 1). Pironetin (PRN) is a naturally occurring plant growth regulator isolated from the culture broth of *Streptomyces* sp. NK10958 [9,10]. PRN is a dihydropyrone derivative and its α,β-unsaturated lactone is absolutely essential for its microtubule inhibitory activity. The alkyl chain and the hydroxyl group at the 7-position are also important for the inhibition of the microtubule dynamic [11]. Early on, PRN was shown to function as an inducer of microtubule disassembly, endowed with marked antitumor properties [12,13]. Detailed investigations of the mode of binding of PRN to α-tubulin have revealed that the drug interacts with Lys352 residue [14] and reacts covalently with Cys316 to destabilize microtubules (Figure 1) [15,16]. Dynamic molecular models of the PRN/α-tubulin complex have been produced [17].

The unique capacity of PRN to interfere with microtubule assembly via α-tubulin binding, coupled with its antitumor properties, have stimulated the search for more potent analogues. Over the past 20 years, synthetic approaches have been explored to produce PRN via different chemical routes [18,19,20,21,22] and to generate simplified PRN derivatives with improved pharmacological properties and metabolic stability [23,24,25,26,27,28,29]. Hybrid molecules combining the scaffold of PRN with other MTAs, such as combretastatin and colchicine, have been designed as well [30,31,32]. Overall, α-tubulin remains an attractive and underexplored target for the design of anticancer agents [7].

Different chemical approaches can be used to design PRN analogues and PRN-inspired derivatives [33]. Alternatively, it is possible to search through the natural product data bank for PRN-like molecules. In this context, our approach has consisted of searching for natural products containing a dihydropyrone scaffold similar to that of PRN and investigating the potential of binding of the identified compounds to the PRN-binding site on α-tubulin using molecular docking. Here we report our discovery of the capacity of the natural products cryptoconcatones to interact with α-tubulin at the PRN site. Structure–binding relationships are discussed.

**Figure 2:**
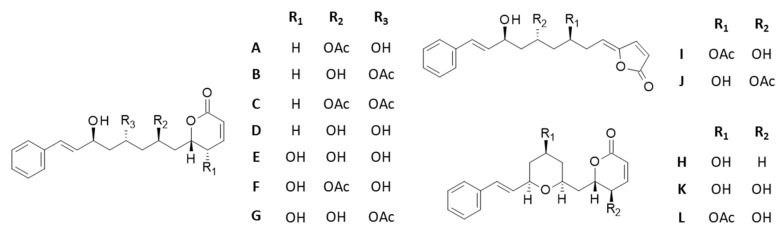
Structures of cryptoconcatones A-L isolated from the leaves and twigs of the tree *Cryptocarya concinna* Hance (Lauraceae).

Twelve cryptoconcatone derivatives, designated cryptoconcatones A-L (Figure 2) were isolated by Yang and co-workers [34,35] from the leaves and twigs of *Cryptocarya concinna* Hance, a tree largely distributed in southeast Asia and used for its robust wood. These compounds contain arylalkenyl α,β-unsaturated δ/γ-lactones and some of them display anti-inflammatory properties [34,35]. Cryptoconcatones K and L have been shown to inhibit proliferation and to induce cytotoxic effects in Huh7 hepatocellular carcinoma cell lines (IC_50_ = 4.5 and 3.9 μM, respectively) [35]. However, these natural products have been little studied thus far and no molecular target has been proposed to explain their cytotoxic properties. Here we reveal the capacity of these compounds to bind to the PRN site of α-tubulin, thus offering a possible explanation for their cytotoxic effects.

## 2. Results

**Figure 3:**
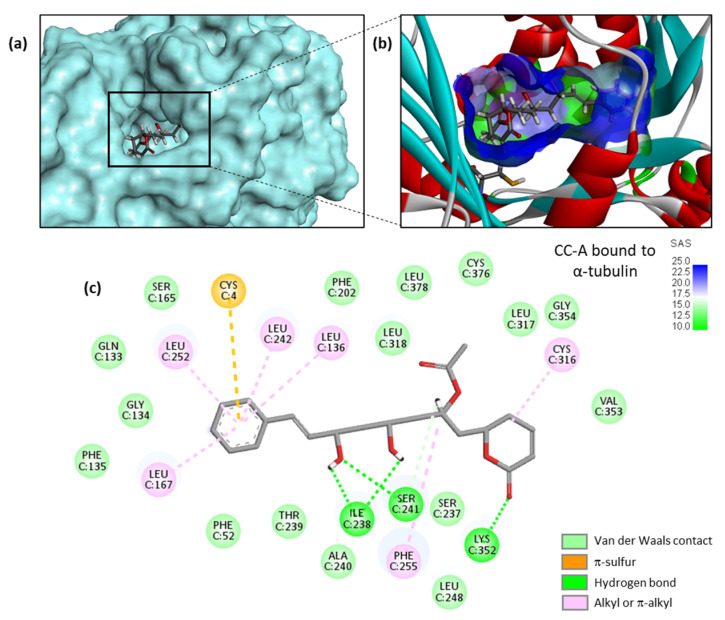
Molecular model of cryptoconcatone A (CC-A) bound to α-tubulin. (**a**) CC-A fits into a cavity of the protein. (**b**) A close-up view of CC-A inserted into the binding cavity, with the solvent-accessible surface (SAS) surrounding the drug binding zone (color code indicated). A ribbon model of α-tubulin is shown, with α-helices (in red) and β-sheets (in cyan). (**c**) Binding map contacts for CC-A bound to α-tubulin (color code indicated).

A high-resolution crystal structure of PRN bound to α/β-tubulin dimer is available (PDB: 5FNV). This structural model has been used to investigate binding of the various cryptoconcatones to the pironetin site, via a molecular docking analysis. There is a narrow but deep cavity in the center of α-tubulin in which the different ligands can insert, with the phenylalkenyl moiety positioned toward the floor of the cavity and the dihydro-α-pyrone moiety toward the opening of the cavity, as represented in Figure 3a with cryptoconcatone A (CC-A). The binding pocket is small but sufficiently deep to accommodate the ligand completely, and remains well accessible to the solvent (Figure 3b). Multiple molecular contacts can be established between the natural product and the protein to stabilize the complex. We could identify up to 26 potential contacts, including 3 H-bonds with the key residues Lys352, Ile238 and Ser241, plus a range of van der Waals, alkyl/π-alkyl and π -sulfur contacts (Figure 3c).

For each compound, a specific model was built and the empirical energy of interaction (ΔE) and free energy of hydration (ΔG) were calculated (Table 1). Interestingly, almost all compounds provided ΔE values more negative than those calculated with the reference PRN. Only CC-K was found to bind poorly to α-tubulin.

**Figure 4:**
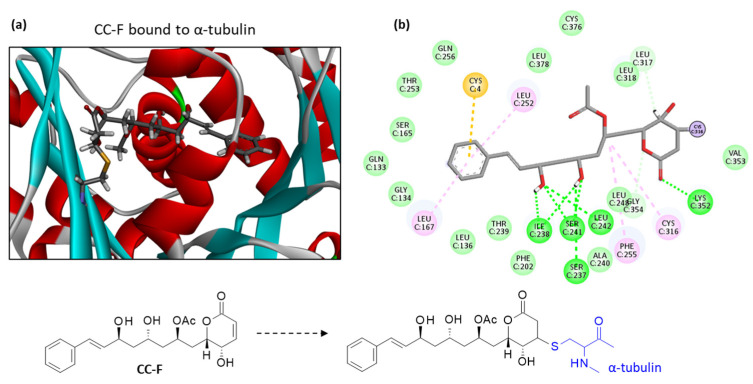
Molecular model of cryptoconcatone F (CC-F) bound to α-tubulin. (**a**) A detailed view of CC-F inserted into the binding cavity. The drug is covalently bound to the protein, with α-helices (in red) and β-sheets (in cyan). (**b**) Binding map contacts for CC-F bound to α-tubulin (color code as in Figure 3). Note that in (**b**), the compound interaction is represented prior to the covalent binding to Cys316. The structures of CC-F free and bound to the Cys316 residue of the protein are represented below the model.

Among the subseries of seven compounds with a similar phenylalkenyl α,β-unsaturated delta-lactone core (CC-A to CC-G), the best two compounds are CC-C and CC-F. This latter compound emerged as the best ligand in this study. CC-F also displays a lower solvation free energy (ΔG) than CC-C. A molecular model of CC-F bound to α-tubulin is presented in Figure 4. The bonding interaction between the Cys316 residue of the protein and the drug can be clearly seen, together with the extended conformation of the ligand within the binding site. Multiple molecular contacts maintain the compound in the cavity (Figure 4b). Compared to CC-A, CC-F interacts with the protein via a greater number of H-bonds, notably with residues Ser-237 and Leu-242, but the two protein-bound drug configurations are very similar. They both present the same key three hydroxyl groups necessary to anchor the drug in the protein cavity. The central -OH (R_3_) is important because its substitution with an acetyl group, as in CC-B, CC-C and CC-G, reduces the binding interaction with the protein. The other -OH group at R_2_ is apparently less essential; its substitution with an acetyl group is not detrimental to the binding interaction, but is favorable. The best binders, CC-A, -C and -G, all present an -OAc substituent at this position R_2_.

**Figure 5:**
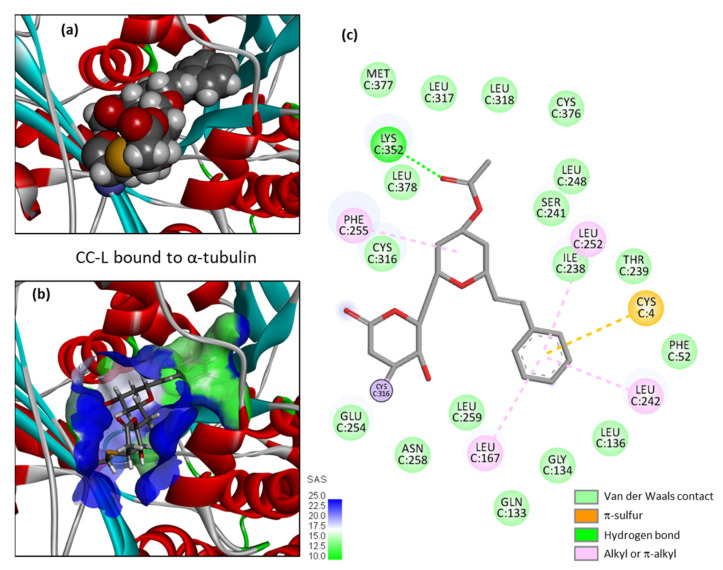
The molecular model of cryptoconcatone L (CC-L) bound to α-tubulin. (**a**) A space-filling (CPK) model of the drug covalently bound to the protein. (**b**) A close-up view of CC-L inserted into the binding cavity, with the solvent-accessible surface (SAS) surrounding the drug binding zone (color code indicated). A ribbon model of α-tubulin is shown, with α-helices (in red) and β-sheets (in cyan). (**c**) Binding map contacts for CC-L bound to α-tubulin (color code indicated).

**Figure 6:**
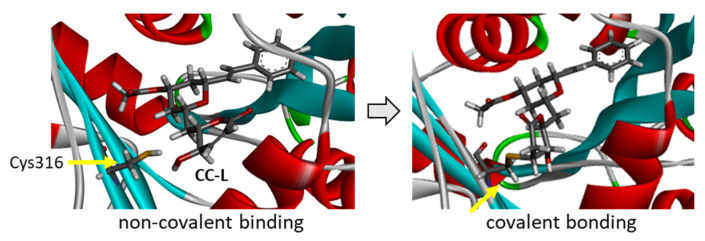
Models of cryptoconcatone L (CC-L) interacting with α-tubulin. The left view shows the ligand inserted into the binding cavity prior to reacting with the proximal thiol group of the Cys316 residue. The right view shows the ligand covalently bound to the Cys316 residue, as highlighted.

Cryptoconcatones I and J (Figure 2), with an arylalkenyl α,β-unsaturated γ-lactone (not δ-lactone, as for all the other cryptoconcatones), display the same capacity to bind to α-tubulin, weaker than that of CC-F but comparable to that of CC-A and CC-G, for example. CC-J is the compound with the lowest solvation free energy in the series but is not the best overall binder. The replacement of the 2-pyranone with a 2-furanone unit is neither an obstacle for protein binding, nor a source of additional interaction. The last three compounds with an arylalkenyl α,β-unsaturated δ-lactone (CC-H, -K, -L) offer interesting observations. The best compound is CC-L with an acetyl group on the central tetrahydropyran ring (at the 4′ position), with a binding capacity almost identical to that of CC-F. Both the empirical energy of interaction (ΔE) and solvation free energy (ΔG) values are similar for the two compounds (Table 1). The deletion of the acetyl group gives a compound with a 4′-OH (CC-K), which is significantly less potent as an α-tubulin binder compared to CC-L. The removal of the hydroxyl on the lactone ring, as in CC-H (R_2_=H), leads to a compound with the weakest protein binding capacity. The two substituents of CC-L (R_1_=OAc and R_2_=OH) play a major role in maintaining the interaction between the compound and α-tubulin, as represented in Figure 5. Upon binding to the PRN site of α-tubulin, the molecule CC-L is positioned with its lactone ring facing the thiol group of the Cys316 residue, at a short distance to allow the covalent reaction. The compound is ideally placed to react with the proximal thiol group, leading easily to the covalent adduct (Figure 6). The same process was observed with CC-F and CC-L, the two best α-tubulin ligands in the cryptoconcatone series.

**Figure 7:**
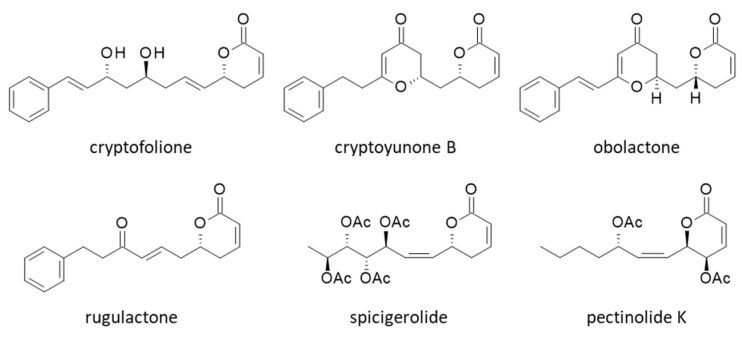
Structures of other pyrone derivatives investigated as α-tubulin binders.

To complete the study, we searched for other natural products bearing an aryl or arylalkenyl δ-lactone as in CC-F to try to identify other α-tubulin binders. We tested five compounds structurally close to CC-F: cryptofolione, cryptoyunone B, obolactone, rugulactone and spicigerolide (Figure 7).

These compounds can be found in *Cryptocarya* species [36,37,38,39]. The ΔE and ΔG values calculated with these compounds are reported in Table 1. None of them provided ΔE values better (more negative) than those calculated with the best two ligands in our series, CC-F and CC-L. However, the lactone spicigerolide revealed a marked capacity to interact with α-tubulin, with an affinity similar to that of CC-A, for example. This compound has been described previously as a cytotoxic agent [40]. Tubulin binding may contribute to the cytotoxic action of spicigerolide.

## 3. Discussion

The evergreen tree *Cryptocarya concinna* Hance is largely distributed in southeast Asia and southern China [41,42]. The wood of the tree is frequently used for housing and furniture making. Many bioactive natural products have been isolated from the plant, notably from the young leaves, which contain phenolic compounds with antioxidant properties [43]. They also contain compounds with antimicrobial and mosquito larvicidal activities [44]. A series of 12 lactone derivatives, called cryptoconcatones A-L, has been isolated from the plant and some of these compounds have revealed interesting biological properties [34,35]. This is the case notably for cryptoconcatone L, which is a cytotoxic molecule, inhibiting proliferation of Huh7 hepatocellular carcinoma cells [35]. The mechanism of action of the natural product has never been investigated. Here we provide computational evidence suggesting that the compound could function as a tubulin binder. Nevertheless, tubulin binding is not the sole parameter to explain the cytotoxicity of the compounds. CC-L emerges as a potent potential tubulin binder, whereas the analogue CC-K exhibits a much weaker tubulin binding profile (at least according to our calculations), but is also a cytotoxic compound [35]. There are other parameters that play a role in the cytotoxic action (drug uptake, excretion, metabolism, etc).

Cryptoconcatone L and its analogue cryptoconcatone F both present a prominent capacity to bind to the pironetin site of α-tubulin. These two compounds have been completely neglected thus far and rarely studied. There are described chemical procedures for the synthesis of cryptoconcatones D, H and I [45,46,47]. However, there are no pharmacological studies defining their molecular target and activities. The present work opens novel perspectives to understand their mode of action. The molecular models of cryptoconcatone/α-tubulin complexes reported here can also facilitate the design of novel analogues targeting the microtubule network. Molecular docking is a useful approach to investigate and propose protein targets and to guide the design of more potent analogues.

The pironetin site of α-tubulin is viewed as a target for the design of anticancer agents. Microtubule inhibitors have been extensively studied, but most of them bind to the vinca-alkaloid or taxane sites on β-tubulin [8]. The pironetin binding site on α-tubulin is considered an unexplored target for cancer therapeutics [7]. There are a few recent studies dedicated to the design of novel pironetin derivatives, such as phenylpironetin analogs [28,29] and functionalized pironetin analogs [25,26,27]. Otherwise, the pironetin scaffold is not frequently used as a template to build anticancer agents. Nevertheless, it is interesting to refer to a study about the properties of 2-pyranone derivatives isolated from *Hyptis* species (Lamiaceae) to bind to the pironetin site of α-tubulin. These compounds, named pectinolides, exert cytotoxic effects toward cancer cells. The best compound in the series, pectinolide K (Figure 7), has shown submicromolar activities against breast, cervix and colon cancer cells in vitro (IC_50_ = 0.5, 0.7 and 0.8 µM, against MCF7, HeLa, and HCT-15 cells respectively) [48]. Based on a molecular docking analysis (similar to the one reported here), the authors concluded that pectinolide K binds to the pironetin site on α-tubulin, using notably H-bonding interaction with residue Lys352. The situation for this pyrone derivative, structurally close to spicigerolide, is totally reminiscent of that described here with cryptoconcatones F and L. The protein binding process implicates Lys352 and Val353, and the respective orientations of the compounds in the pironetin site are very similar (but according to our calculation, pectinolide K is much less prone to α-tubulin binding than CC-F (ΔE = −48.9 and −74.1 kcal/mol, respectively)). Based on their model, the authors designed a series of 6-heptyl-5,6-dihydro-2H-pyran-2-ones targeting α-tubulin [48]. In conclusion, our work, entirely consistent with other observations, attests that the cryptoconcatone and pironetin scaffolds can be further exploited to design anticancer agents. There exists a variety of 5,6-dihydro-α-pyrones in plants, such as monticolides [49], pulchrinervialactone [50], cryptorigidifoliols [51], cryptomoscatones [36] and others. All these compounds should be tested as modulators of microtubule dynamics.

## 4. Materials and Methods

### 4.1. Molecular Structures and Software

The three-dimensional structure of pironetin bound to α-tubulin was retrieved from the Protein Data Bank (www.rcsb.org, accessed on 23 December 2022) under the PDB code 5FNV. The structure has been determined by X-ray diffraction with a good resolution (2.61 Å) [15]. Docking experiments were performed using the GOLD software (GOLD 5.3 release, Cambridge Crystallographic Data Centre, Cambridge, UK). Molecular graphics and analyses were performed using Discovery Studio Visualizer, Biovia 2020 (Dassault Systèmes BIOVIA Discovery Studio Visualizer 2020; San Diego, Dassault Systèmes, 2020).

### 4.2. In Silico Molecular Docking Procedure

The process used includes the following five steps:

(1) Monte Carlo (MC) conformational search of the ligand using the BOSS (Biochemical and Organic Simulation System) software, freely available to academic users. The structure of the ligand was optimized using a classical MC conformational search procedure, as described in BOSS [52]. A conformational analysis was performed to define the best starting geometries for each compound. An energy minimization was carried out to identify all minimum-energy conformers, leading to the identification of a unique conformer for the free ligand. Within BOSS, MC simulations were performed in the constant-temperature and constant-pressure ensemble (NPT).
(2) Evaluation of the free energy of hydration for the chosen structure of the ligand. The molecular mechanics/generalized Born surface area (MM/GBSA) procedure was used to evaluate the free energies of hydration (ΔG) (Jorgensen and Tirado-Rives, 2005) [53]. MC search and computation of ΔG were performed within BOSS using the xMCGB script according to procedures given in references [53,54]. The best ligand structure was then used in the docking procedure.
(3) Definition of the α-tubulin-ligand site of interaction. The pironetin binding site was defined as the binding site for all α-pyrone derivatives tested. With the 5FNV structure, based on shape complementarity criteria, the flexible amino acids are Phe135, Phe202, Leu248, Leu252, Phe255, Gln256, Leu259, Cys316, Lys352, and Leu378. Shape complementarity and geometry considerations favor a docking grid centered in the volume defined by the central amino acid. Within the binding site, the side chains of the specific amino acids were considered fully flexible during docking.
(4) Docking procedure using GOLD. In our typical docking process, 100 energetically reasonable poses (according to the ChemPLP scoring function) are retained while searching for the correct binding mode of the ligand. The decision to maintain a trial pose is based on ranked poses, using the PLP fitness scoring function (which is the default in GOLD version 5.3 used here) [55]. Six poses are kept. The empirical potential energy of the interaction ΔE for the ranked complexes was evaluated using the simple expression ΔE(interaction) = E(complex) − [E(protein) + E(ligand)]. Calculations of the final energy are performed on the basis of the SPASIBA spectroscopic force field. The corresponding parameters are derived from vibrational wavenumbers obtained in the infrared and Raman spectra of a large series of compounds including organic molecules, amino acids, saccharides, nucleic acids and lipids.
(5) Validation using the SPASIBA force field. This last step is considered essential to define the best protein–ligand structure. The spectroscopic SPASIBA (Spectroscopic Potential Algorithm for Simulating Biomolecular conformational Adaptability) force field has been specifically developed to provide refined empirical molecular mechanics force field parameters [56]_._ SPASIBA empirical energies of interaction are calculated as described [57,58]. SPASIBA (integrated into CHARMM) [59] has been shown to be excellent in reproducing crystal phase infrared data. The same procedure was used to establish molecular models for the various drug–protein complexes.

## 5. Conclusions

The dihydropyrone compounds cryptoconcatones isolated from the tree *Cryptocarya concinna* Hance can bind to tubulin dimer and target the pironetin site on α-tubulin. The best binders are cryptoconcatones F and L. These compounds warrant further investigation as potential modulators of tubulin dynamics and as anticancer agents.

## Figures and Tables

**Table 1:** Calculated potential energy of interaction (ΔE) and free energy of hydration (ΔG) for the interaction of the indicated natural products with tubulin.

Compounds	ΔE (kcal/mol)	ΔG (kcal/mol)
Pironetin	−57.32	−24.20
Cryptocontanone A	−67.80	−25.00
Cryptocontanone B	−63.10	−24.25
Cryptocontanone C	−70.30	−26.25
Cryptocontanone D	−60.70	−27.50
Cryptocontanone E	−65.40	−22.00
Cryptocontanone F	−74.15	−27.60
Cryptocontanone G	−66.00	−24.40
Cryptocontanone H	−57.70	−23.30
Cryptocontanone I	−66.65	−29.10
Cryptocontanone J	−66.75	−30.75
Cryptocontanone K	−52.30	−22.85
Cryptocontanone L	−73.40	−26.50
Cryptofolione	−56.20	−21.75
Cryptoyunone B	−60.20	−19.80
Obolactone	−52.50	−18.50
Rugulactone	−59.50	−16.80
Spicigerolide	−67.70	−21.00

## Data Availability

Data is contained within the article.
